# Outpatient administration of CAR T-cell therapy: a focused review with recommendations for implementation in community based centers

**DOI:** 10.3389/fimmu.2024.1412002

**Published:** 2024-05-08

**Authors:** Ariel Perez, Tiba Al Sagheer, George R. Nahas, Yuliya P. L. Linhares

**Affiliations:** Miami Cancer Institute, Baptist Hospital of Miami, Miami, FL, United States

**Keywords:** chimeric antigen receptor (CAR T), outpatient setting, community based, cellular therapy, implementation

## Abstract

Chimeric Antigen Receptor T-cell (CAR-T) therapy has transformed the treatment landscape for hematological malignancies, showing high efficacy in patients with relapsed or refractory (R/R) disease and otherwise poor prognosis in the pre-CAR-T era. These therapies have been usually administered in the inpatient setting due to the risk of cytokine release syndrome (CRS) and immune effector cell-associated neurotoxicity syndrome (ICANS). However, there is a growing interest in the transition to outpatient administration due to multiple reasons. We review available evidence regarding safety and feasibility of outpatient administration of CD19 targeted and BCMA targeted CAR T-cell therapy with an emphasis on the implementation of outpatient CAR-T programs in community-based centers.

## 1 Introduction

Chimeric Antigen Receptor T-cell (CAR-T) therapy has transformed the treatment landscape for hematological malignancies, showing high efficacy in patients with relapsed or refractory (R/R) disease and otherwise poor prognosis in the pre-CAR-T era. In the last seven years, the United States Federal Drug Administration (FDA) has approved six CAR-T products for several hematologic malignancies, such as large B-cell lymphomas (LBCL), B-cell acute lymphoblastic leukemia (B-ALL), mantle cell lymphoma (MCL), follicular lymphoma (FL), multiple myeloma (MM) and recently chronic lymphocytic leukemia/small lymphocytic lymphoma (CLL/SLL) (1).

Historically, these therapies have been administered in the inpatient setting due to the risk of cytokine release syndrome (CRS) and immune effector cell-associated neurotoxicity syndrome (ICANS). However, there is now an interest in investigating the safety and feasibility of outpatient administration of CAR-T therapy in different settings. To do so several factors must be considered, including efficacy and safety profile of each product, patient and disease characteristics, center infrastructure, logistic aspects, economic implications, and regulatory considerations. The American Society for Transplantation and Cellular Therapy convened an expert panel to review current evidence, survey cellular therapy programs regarding outpatient administration of these products and provide guidance on this matter (2).

The evolution towards outpatient CAR T-cell therapy is motivated by the pursuit of optimizing resource utilization, reducing healthcare costs, increasing patient satisfaction and convenience. However, this paradigm shift introduces complexities such as the need for frequent outpatient monitoring including telehealth visits and potentially using wearable devices for vital signs monitoring. The need to strike a balance between patient safety and successful outcomes with implementation logistics and resource utilization becomes imperative in this dynamic landscape.

The safety and feasibility of outpatient administration of CAR T-cell products have been shown by several groups, mostly in academic centers. University-based programs have established cellular therapy programs for blood and marrow transplantation (BMT), with the added advantage of clinical CAR-T experience acquired through participation in registrational trials and early implementation of standard of care (SoC) programs once CAR- T-cells became commercially available. Administration in non-academic centers or hematology practices affiliated with community hospitals represents a challenge. Products with a predictable time frame for immune effector cell toxicities and/or with lower toxicity profiles can be administered safely as outpatient in most institutions. This review aims to offer a comprehensive analysis of the current state, challenges, and advancements in outpatient CAR-T therapy, with a focus on its application in community-based centers with cellular therapy programs.

## 2 CD19 targeted CAR T-cell therapy for B-cell malignancies

Four commercial CD19 targeted CAR-T products have been approved for the treatment of R/R B-cell malignancies. Axicabtagene ciloleucel (axi-cel) received approval for relapsed or refractory Large B-cell Lymphoma (LBCL) and Follicular Lymphoma (FL), tisagenlecleucel (tisa-cel) received approval for LBCL, FL and B-cell ALL (in patients up to 25 years of age), lisocabtagene maraleucel (liso-cel) for LBCL and CLL/SLL, while brexucabtagene autoleucel (brexu-cel) is approved for R/R mantle cell lymphoma (MCL) and B-cell ALL (3–11). CAR-T toxicity profile is variable and is partially dependent on the type of co-stimulatory domain used in the CAR construct, with higher rates of neurological toxicities and CRS observed with CARs containing CD28 versus 4-1BB costimulatory domains (12). Most registrational studies for B-cell malignancies mandated obligatory hospital stay for the monitoring and management of CAR-T toxicities including CRS and ICANS. Upon CAR-T approval, the standard approach has been to hospitalize patients for infusion and toxicity monitoring. This creates administrative challenges due to increased inpatient bed utilization and staffing demands. Additionally, a planned prolonged hospital stay represents an emotional and logistical burden to patients and caretakers. Strategies for safe outpatient administration of CAR-T therapies are needed to improve accessibility. Successful outpatient administration and management of patients treated with tisa-cel, liso-cel, axi-cel, and brexu-cel have been previously reported (13–16).

Results of a US Cell Therapy Consortium analysis for LBCL patients showed outpatient tisa-cel administration was feasible and safe. In the outpatient group less than half (45%) of tisa-cel recipients required an unplanned admission. The median length of stay for patients who had an unplanned admission due to toxicities was shorter (5 days vs 13 days) when compared to the inpatient group (16). Another large database analysis, this one from the Center for International Blood & Marrow Transplant Research (CIBMTR), showed real world liso-cel outcomes paralleled those of the TRANSCEND trial with an overall favorable toxicity profile. The incidence of grade ≥ 3 CRS and ICANS were 3% and 11%, respectively, while 48% and 70% of patients did not experience any CRS or ICANS (13). These findings support the feasibility of outpatient liso-cel administration. This was confirmed by the OUTREACH (NCT03744676) trial which evaluated liso-cel in patients with third line or later R/R LBCL treated at US community sites with outpatient monitoring. Inpatient monitoring was allowed in the study at investigator’s discretion. Among 82 patients treated with liso-cel, 70% were monitored as outpatients. The need for inpatient monitoring was mostly due to unfavorable disease characteristics. Any grade CRS reported in 40% of patients; with no patients experiencing grade ≥3 CRS. In terms of neurological events, 29% of patients experienced any grade ICANS with 10% experiencing GR ≥ 3. Tocilizumab and/or corticosteroids were administered to 29% of patients. In outpatients, 37% experienced any grade CRS and 28% any grade ICANS. Hospitalization was not necessary in 25% of patients who started with the outpatient infusion and 32% were hospitalized within 4 days. Initial hospitalization lasted a median of 6 days (range, 1–28) for outpatients versus 15 days (range, 3–31) for inpatients. The OUTREACH study showed outpatient liso-cel administration is feasible and safe outside of academic center settings (14).

The University of Pennsylvania group reported their outpatient experience with tisa-cel in R/R non-Hodgkin lymphoma (NHL). After lymphodepletion, patients were evaluated for the presence of bulky disease, organ dysfunction or increase in symptoms. Those without risk factors received outpatient tisa-cel with frequent clinic visits for monitoring. A total of 68 patients were treated with outpatient tisa-cel. Any grade CRS rate was 40.3%, with no reported grade 3-5 CRS events. Hospitalization was required in 19.4% of patients within 72 hours, and in 36.1% within 30 days of product infusion. The median length of hospital stay was 5 days. This analysis demonstrated the safety and feasibility of outpatient tisa-cel administration, with 64% of patients not requiring hospitalization (17).

Axi-cel and brexu-cel have some of the highest response rates but carry higher risks of CRS and ICANS. Dholaria and colleagues reported a series of 13 patients treated with axi-cel and brexu-cel (LBCL, n=9; MCL, n=4) in the outpatient setting (15). Relevant staff and caregiver training was implemented, as well as telemedicine visits for remote outpatient monitoring. Criteria for outpatient administration included tumor sum of product diameters (SPD) less than 1000 mm^2^ and minimal comorbidities. Patients were monitored with twice daily in-person visits and one overnight remote visit via telemedicine through day 14 post- CAR-T infusion. The median time to hospitalization was 96 hours with a median inpatient stay of 7 days. Most (92%) patients experienced CRS, and more than half (54%) experienced neurological toxicities. Hospitalization was not necessary in 3 (23%) patients. This study highlights the possibility of avoiding hospitalizations in selected patients receiving CAR-T products with CD28 costimulatory domain. The frequency of clinical follow up visits (both in-person and telehealth) may not be feasible in all institutions due to staffing demands, resources, and patient or caretaker limitations. The Medical University of South Carolina group reported their real-world experience with outpatient CAR-T. Of the 32 patients who received outpatient infusion, hospitalization was not required through day +30 for 4 (12.5%) patients (18).

## 3 BCMA targeted CAR T-cell therapy for multiple myeloma

Two B-cell maturation antigen (BCMA) directed CAR-T products, idecabtagene vicleucel (ide-cel) and ciltacabtagene autoleucel (cilta-cel) are approved for patients with R/R multiple myeloma (RRMM). Despite their efficacy in heavily pre-treated populations, accessibility remains a challenge. The registrational study for ide-cel required the first 10 patients to remain hospitalized for a minimum of 7 days following product administration, thereafter, outpatient management of additional patients was an option (NCT03361748). The registrational study for cilta-cel (NCT03548207) mandated hospitalization of the first 6 patients for at least 2 weeks after receiving cilta-cel infusion. Patients who received cilta-cel outpatient were asked to remain within 1 hour of the study site for 2 weeks after cell infusion (19, 20).

Fortunately, as our understanding of CAR-T toxicities, prophylaxis and management strategies continue to improve the field is moving towards outpatient administration and hospitalization either at defined timepoints or once immune effector-cell related toxicities occur. For example, the feasibility of outpatient cilta-cel is currently being explored in the phase II CARTITUDE-2 MMY2003 (NCT04133636) and the phase III CARTITUDE-5 MMY3004 (NCT04923893) studies. Lymphodepleting chemotherapy and cell product infusion are administered in the outpatient setting with planned hospitalization on day +5. Deferring hospitalization until day +5 is reasonable given that the median times to onset of CRS and ICANS for this product are 7 and 8 days, respectively.

For CAR-T products like ide-cel, where toxicity onset is earlier, alternative strategies are needed. The median onset of CRS after ide-cel infusion is 1 day and the median onset of ICANS is 2 days. Bansal and colleagues reported the results of outpatient administration of several CAR-T products including ide-cel and cilta-cel. Patients were admitted based on certain clinical or laboratory findings like presence of fever, elevated C- reactive protein within 24-hour or less doubling time, or new neurologic symptoms. All three patients in the outpatient ide-cel group were admitted. For cilta-cel, 17 of 22 patients were admitted. While these numbers remain small, there does seem to be a potential signal that outpatient administration of BCMA products is feasible (21). Thus far outpatient CAR-T administration has not been shown to worsen overall safety outcomes. Lastly, it should be noted that median length of hospital stay was relatively short at 4 and 5 days for ide-cel and cilta-cel, respectively.

In an effort to improve detection and early intervention of CAR-T related toxicities, the use of wearable technology has been implemented by some institutions. These devices can remotely detect changes in temperature, pulse, oxygen saturation, and respiratory rate. Rajeeve et al. report the results of an investigator initial trial that enrolled 14 patients evaluating the feasibility of using wearable devices (WD) for detecting CRS following autologous CAR-T therapy in RRMM. Most notable in this study was the excellent inter-observer correlation (95% CI) = 0.952 (0.901-0.977). Additionally, CRS was detected by threshold temperature method approximately 3 hours earlier than SoC nursing vital signs (22). The authors concluded that reliable CRS detection by WD may support the transition from inpatient to outpatient administration of cellular therapies. Further efforts are underway in order to further explore this promising and convenient platform.

## 4 Patient selection for outpatient CAR-T

Adequate patient selection is essential for successful outpatient CAR-T administration. The ASTCT expert panel opinion outlined several factors that should be considered when evaluating a patient for outpatient CAR-T therapy (2). These factors can be grouped into clinical and logistical aspects. Age, performance status, comorbidities, organ function, and disease characteristics are among the most relevant clinical factors to consider. The specific criteria to consider outpatient versus inpatient may differ between treatment centers and cellular therapy physicians. Logistical aspects for outpatient administration include proximity to the treatment center, around the clock caregiver support and 24/7 access to qualified healthcare personal for direct admission and emergent management of complications.

Product selection for outpatient CAR T-cell therapy administration is also dependent on the toxicity profile of each CAR-T product and the time to onset of CRS and ICANS. Certain products exhibit a rapid onset of toxicities (axi-cel, brexu-cel and ide-cel) after product infusion, while others exhibit a later CRS and ICANS onset (liso-cel, cilta-cel, tisa-cel). The time to onset and the rates of severe CRS/ICANS can inform decisions regarding outpatient CAR-T infusion and help guide planned admissions at specified timepoints.

## 5 Discussion

In non-academic settings, CAR-T products with a rapid toxicity onset or higher rates of Grade >=3 CRS/ICANS may be administered in a hospital environment for close monitoring in the post infusion period, while the late onset or lower toxicity agents allow for easier planning of outpatient infusion and follow-up, tailed by a planned admission for monitoring closer to the time point of toxicity emergence or at the time of toxicity occurrence, therefore shortening the overall hospital stay duration and improving bed space utilization.

Community based centers with more limited resources may favor inpatient administration of products with higher toxicity rates and early onset of CRS/ICANS due to staffing limitations, lack of timely after-hours admission pathway, and lack of dedicated 24/7 emergency units with adequately trained personal. Moreover, the implementation of preventative interventions such as corticosteroids or anakinra can exert influence on the time of onset and severity of CRS and ICANS (23–25). For example, the prophylactic administration of corticosteroids, as observed in cohort 6 of the ZUMA-1 trial, resulted in a delay in CRS onset to 5 days compared to the earlier onset observed in cohort 1 (23).

If the objective is to administer CAR-T therapy in the outpatient setting, it may be prudent to consider widespread prophylactic strategies along with implementation of safety measures across the healthcare system with the aim of mitigating and delaying severe toxicities. For community-based outpatient CAR-T programs it is essential to reach providers in the emergency department (ED) and other urgent services within the community network and create awareness of the need for quick intervention in cellular therapy recipients. Electronic Medical Record (EMR) labels and patient chart alerts for CAR-T recipients that include contact numbers for the cell-therapy team are useful and can provide a safety net to avoid delays in care. Caregiver support is also a critical component and education must include provider contact information and vital signs self-monitoring (26). If wearable devices are being used the clinical team must ensure caretakers are familiar with their role in detection of vital signs changes.

**Figure 1:**
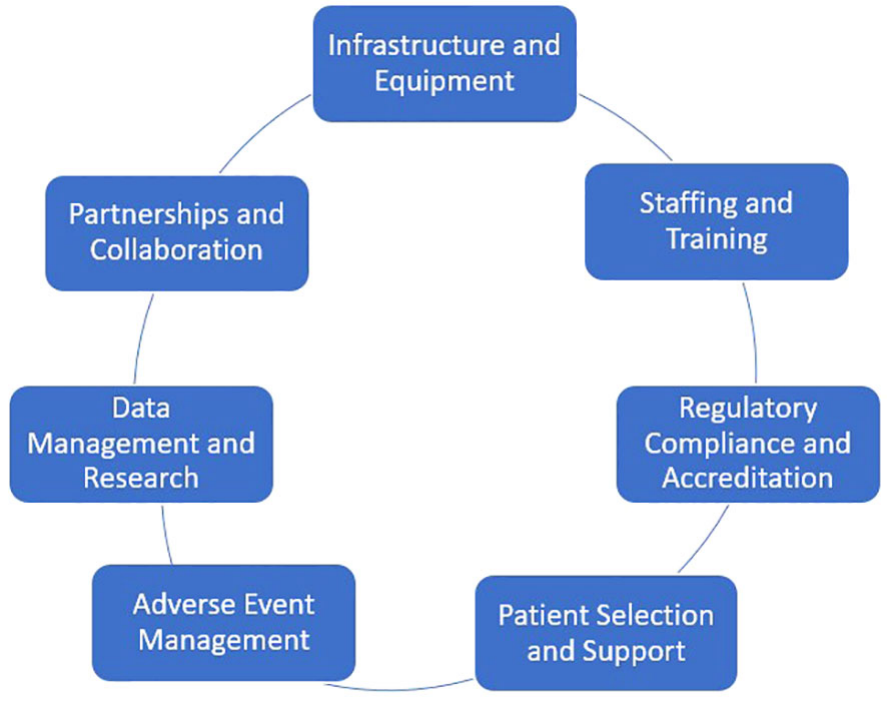
Key components of a CAR-T program in community-based centers.

Establishing an outpatient CAR T-cell therapy program in a non-academic setting is a complex but achievable goal. A successful program requires careful planning, significant resources, and adherence to regulatory standards. Some of the key components needed to establish such a program are shown in **Figure 1**. A detailed description of each component is provided here:

### 5.1 Infrastructure and equipment

#### 5.1.1 Facilities

Dedicated spaces with the necessary infrastructure to perform leukapheresis, administer cell therapy and monitor patients for adverse reactions. Centers with pre-existing BMT programs have an easier path to implementation.

#### 5.1.2 Laboratory services

Access to a laboratory capable of handling pre-CAR-T testing, ongoing monitoring, and emergent need for services.

### 5.2 Staffing and training

#### 5.2.1 Specialized staff

A team of healthcare professionals trained in CAR T-cell therapy administration, including hematologists, advance practice providers, nurses, pharmacists, coordinators, and support staff. Training should cover the unique side effects and management protocols specific to CAR-T therapy.

#### 5.2.2 Continuous education

Ongoing training programs to keep staff updated on the latest developments, protocols, and safety measures. Extending education to other providers and staff outside of the Hematology and Cell Therapy programs is essential to increase awareness and enhance communication in centers affiliated with community hospitals.

### 5.3 Regulatory compliance and accreditation

#### 5.3.1 Certification

Obtaining certification from regulatory bodies, such as the FDA, which often requires adherence to specific guidelines for facilities and staff involved in administering CAR-T therapy. In the US all CAR-T products are available under a REMS (Risk Evaluation and Mitigation Strategy) program to ensure safe administration. REMS requirements typically include elements aimed at educating healthcare providers and patients about potential risks and appropriate use. These encompass training programs for healthcare professionals, as well as patient education materials outlining the treatment’s benefits, risks, and necessary precautions. REMS for CAR-T products also involve protocols for monitoring and managing adverse events, ensuring timely reporting and intervention once immune effector -cell related toxicities occurs.

#### 5.3.2 Accreditation

Pursuing accreditation from professional organizations, such as the Foundation for the Accreditation of Cellular Therapy (FACT), which can help ensure adherence to best practices in cellular therapy.

### 5.4 Patient selection and support

#### 5.4.1 Eligibility criteria

Establishing criteria for patient eligibility based on the current indications for CAR-T and considering the patient’s overall health and ability to tolerate the therapy. Guidelines for inpatient vs outpatient administration and established care pathways should be available.

#### 5.4.2 Support services

Providing comprehensive patient support, including education about CAR-T therapy, financial counseling, psychological support, and long-term follow-up care.

### 5.5 Adverse event management

#### 5.5.1 Protocols for side effects

Developing detailed protocols for the management of common CAR-T therapy side effects, such as CRS, ICANS, hematotoxicity and infectious complications.

#### 5.5.2 Emergency care

Ensuring rapid access to emergency services and intensive care units capable of managing severe adverse reactions.

### 5.6 Data management and research

#### 5.6.1 Patient registry

Keeping detailed records of treatments and outcomes to contribute to the broader knowledge base of CAR T-cell therapy safety and efficacy. Encourage participation and reporting to CIBMTR or similar collaborative registry-based research efforts.

### 5.7 Partnerships and collaboration

#### 5.7.1 Collaboration with academic centers

Forming partnerships with academic centers and industry leaders in CAR-T therapy for knowledge exchange, research participation, and referral networks.

In summary, available data supports the safety and feasibility of outpatient CAR-T administration with the caveat of a majority of the studies being conducted in large academic centers, where resources are readily available. Community-based programs need to overcome additional challenges for implementation of successful CAR T-cell programs and transition to outpatient administration in a safe manner. Establishing pathways that ensure quick and efficient transitions of care between outpatient and inpatient is essential. Telehealth visits and WD are useful tools, but their widespread implementation may be limited due to lack of funding and staffing demands. Outpatient CAR-T therapy programs in non-academic settings will continue to expand as the indications and spectrum of commercially available products broadens. When evaluating the financial aspects of administering CAR-T in an outpatient setting, it’s crucial to understand the factors that impact overall costs. One key element is the potential for reduced acquisition costs, a benefit that is relevant under the 340B Drug Pricing Program available in the US (27). This program allows institutions to purchase medications at discounted prices for outpatient administration. Moreover, Medicare reimbursement policies and patient status determination play a critical role in financial considerations. For instance, if a patient needs to be admitted as inpatient to the hospital during the 72 hours following product infusion, the associated costs are transferred to the inpatient setting and covered under a Diagnosis-Related Group (DRG) payment system (28). This results in lower reimbursement because DRG payments are typically fixed amounts based on the diagnosis and treatment provided, rather than reflecting the actual cost of the therapy. A first step to mitigate these challenges could be selecting CAR-T products with later onset of toxicities where it is feasible to complete lymphodepletion and cell infusion as outpatient, and then admitting pre-emptively on or after day +4. This approach can help US community-based centers with limited resources for afterhours admissions ensure patients receive timely and appropriate care for CAR-T related complications, while optimizing costs. Establishing an outpatient CAR-T program is both challenging and rewarding. It not only requires significant upfront investment and ongoing dedication to excellence but also provides an invaluable service to patients by making life-saving treatments more accessible.

## Author contributions

AP: Writing – original draft, Writing – review & editing. TS: Writing – original draft, Writing – review & editing. GN: Writing – original draft, Writing – review & editing. YL: Writing – original draft, Writing – review & editing.

## References

[1] Food and Drug Administration. Approved Cellular and Gene Therapy Products (2019). U.S. Food and Drug Administration. Available online at: https://www.fda.gov/vaccines-blood-biologics/cellular-gene-therapy-products/approved-cellular-and-gene-therapy-products (Accessed March 31, 2024).

[2] Oluwole OO Dholaria B Knight TE Jain T Locke FL Ramsdell L. Chimeric antigen receptor T-cell therapy in the outpatient setting: an expert panel opinion from the American society for transplantation and cellular therapy. Transplant Cell Ther. (2023) 30(2):131–42. doi: 10.1016/j.jtct.2023.11.008 37951502

[3] Locke FL Miklos DB Jacobson CA Perales MA Kersten MJ Oluwole OO. Axicabtagene ciloleucel as second-line therapy for large B-cell lymphoma. New Engl J Med. (2022) 386:640–54. doi: 10.1056/NEJMoa2116133 34891224

[4] Jacobson CA Chavez JC Sehgal AR William BM Munoz J Salles G. Axicabtagene ciloleucel in relapsed or refractory indolent non-Hodgkin lymphoma (ZUMA-5): a single-arm, multicentre, phase 2 trial. Lancet Oncol. (2022) 23:91–103. doi: 10.1016/S1470-2045(21)00591-X 34895487

[5] Laetsch TW Maude SL Rives S Hiramatsu H Bittencourt H Bader P. Three-year update of tisagenlecleucel in pediatric and young adult patients with relapsed/refractory acute lymphoblastic leukemia in the ELIANA trial. J Clin Oncol. (2023) 41:1664. doi: 10.1200/JCO.22.00642 36399695 PMC10022844

[6] Schuster SJ Tam CS Borchmann P Worel N McGuirk JP Holte H. Long-term clinical outcomes of tisagenlecleucel in patients with relapsed or refractory aggressive B-cell lymphomas (JULIET): a multicentre, open-label, single-arm, phase 2 study. Lancet Oncol. (2021) 22:1403–15. doi: 10.1016/S1470-2045(21)00375-2 34516954

[7] Fowler NH Dickinson M Dreyling M Martinez-Lopez J Kolstad A Butler J. Tisagenlecleucel in adult relapsed or refractory follicular lymphoma: the phase 2 ELARA trial. Nat Med. (2022) 28(2):325–32. doi: 10.1038/s41591-021-01622-0 34921238

[8] Abramson JS Solomon SR Arnason J Johnston PB Glass B Bachanova V. Lisocabtagene maraleucel as second-line therapy for large B-cell lymphoma: primary analysis of the phase 3 TRANSFORM study. Blood. (2023) 141:1675–84. doi: 10.1182/blood.2022018730 PMC10646768 36542826

[9] Siddiqi T Maloney DG Kenderian SS Brander DM Dorritie K Soumerai J. Lisocabtagene maraleucel in chronic lymphocytic leukaemia and small lymphocytic lymphoma (TRANSCEND CLL 004): a multicentre, open-label, single-arm, phase 1–2 study. Lancet. (2023) 402:641–54. doi: 10.1016/S0140-6736(23)01052-8 PMC11753452 37295445

[10] Wang M Munoz J Goy A Locke FL Jacobson CA Hill BT. KTE-X19 CAR T-cell therapy in relapsed or refractory mantle-cell lymphoma. New Engl J Med. (2020) 382:1331–42. doi: 10.1056/NEJMoa1914347 PMC7731441 32242358

[11] Shah BD Ghobadi A Oluwole OO Logan AC Boissel N Cassaday RD. KTE-X19 for relapsed or refractory adult B-cell acute lymphoblastic leukaemia: phase 2 results of the single-arm, open-label, multicentre ZUMA-3 study. Lancet. (2021) 398:491–502. doi: 10.1016/S0140-6736(21)01222-8 34097852 PMC11613962

[12] Cappell KM Kochenderfer JN. A comparison of chimeric antigen receptors containing CD28 versus 4-1BB costimulatory domains. Nat Rev Clin Oncol. (2021) 18:715–27. doi: 10.1038/s41571-021-00530-z 34230645

[13] Palomba ML Crombie JL Nastoupil LJ Andreadis C Isufi I Hunter B. Multicenter, real-world study in patients with R/R large B-cell lymphoma (LBCL) who received lisocabtagene maraleucel (liso-cel) in the United States (US). Transplant Cell Ther. (2024) 30:S40–1. doi: 10.1016/j.jtct.2023.12.071

[14] Linhares Y Freytes C Cherry M Bachier C Maris M Hoda D. CT-045 primary results from OUTREACH: A phase II study of lisocabtagene maraleucel (Liso-cel) administered in the community setting as outpatient or inpatient treatment in patients with relapsed or refractory (R/R) large B-cell lymphoma (LBCL). Clin Lymphoma Myeloma Leukemia. (2023) 23:S514–5. doi: 10.1016/S2152-2650(23)01482-9

[15] Dholaria B Mehraban N Baer B Long N Jayani RV Byrne MT. Feasibility of outpatient administration of axicabtagene ciloleucel and brexucabtagene autoleucel using telemedicine tools: The Vanderbilt experience. Br J haematol. (2022) 198:1073–5. doi: 10.1111/bjh.18339 35765247

[16] Ahmed N Wesson W Mushtaq MU Porter DL Nasta SD Brower J. Patient characteristics and outcomes of outpatient tisagenlecleucel recipients for B cell non-Hodgkin lymphoma. Transplant Cell Ther. (2023) 29:449–e1. doi: 10.1016/j.jtct.2023.04.019 PMC11027185 37120134

[17] Nasta SD Hughes ME Namoglu EC Garfall A DiFilippo H Ballard HJ. Outcomes of tisagenlecleucel in lymphoma patients with predominant management in an ambulatory setting. Clin Lymphoma Myeloma Leukemia. (2022) 22:e730–7. doi: 10.1016/j.clml.2022.04.012 PMC10965010 35595619

[18] McGann M Davis JA Gaffney KJ Smith D Edwards K Hess BT. Real-world experience and optimization of outpatient chimeric antigen receptor T cell therapy. Transplant Cell Ther. (2022) 28:583–5. doi: 10.1016/j.jtct.2022.06.021 35781100

[19] Munshi NC Anderson LD Jr. Shah N Madduri D Berdeja J Lonial S. Idecabtagene vicleucel in relapsed and refractory multiple myeloma. New Engl J Med. (2021) 384:705–16. doi: 10.1056/NEJMoa2024850 33626253

[20] Berdeja JG Madduri D Usmani SZ Jakubowiak A Agha M Cohen AD. Ciltacabtagene autoleucel, a B-cell maturation antigen-directed chimeric antigen receptor T-cell therapy in patients with relapsed or refractory multiple myeloma (CARTITUDE-1): a phase 1b/2 open-label study. Lancet. (2021) 398(10297):314–24. doi: 10.1016/S0140-6736(21)00933-8 34175021

[21] Bansal R Paludo J Hathcock M Spychalla M Khurana A Hampel P. (2023). Outpatient practice pattern for recently approved CAR-T in patients with lymphoma and multiple myeloma. Blood [Internet]. 142(Supplement 1):253–3. Available at: https://ashpublications.org/blood/article/142/Supplement%201/253/502606/Outpatient-Management-of-CAR-T-and-Teclistamab-for.

[22] Rajeeve S Wilkes M Zahradka N Serebyrakova K Kappes K Jackson H. Early detection of CRS after CAR-T therapy using wearable monitoring devices: Preliminary results in relapsed/refractory multiple myeloma (RRMM). Journal of Clinical Oncology. 41(16_suppl):e13626–6. doi: 10.1200/JCO.2023.41.16_suppl.e13626

[23] Oluwole OO Bouabdallah K Muñoz J De Guibert S Vose JM Bartlett NL. Prophylactic corticosteroid use in patients receiving axicabtagene ciloleucel for large B-cell lymphoma. Br J haematol. (2021) 194:690–700. doi: 10.1111/bjh.17527 34296427 PMC8457222

[24] Park JH Nath K Devlin SM Sauter CS Palomba ML Shah G. CD19 CAR T-cell therapy and prophylactic anakinra in relapsed or refractory lymphoma: phase 2 trial interim results. Nat Med. (2023) 29:1710–7. doi: 10.1038/s41591-023-02404-6 PMC11462637 37400640

[25] Strati P Jallouk A Deng Q Li X Feng L Sun R. A phase 1 study of prophylactic anakinra to mitigate ICANS in patients with large B-cell lymphoma. Blood Advances. (2023) 7:6785–9. doi: 10.1182/bloodadvances.2023010653 PMC10692290 37389847

[26] Alexander M Culos K Roddy J Shaw JR Bachmeier C Shigle TL. Chimeric antigen receptor T cell therapy: a comprehensive review of clinical efficacy, toxicity, and best practices for outpatient administration. Transplant Cell Ther. (2021) 27:558–70. doi: 10.1016/j.jtct.2021.01.014 33910041

[27] Health Resources & Services Administration. 340B Drug Pricing Program (2017). Official web site of the U.S. Health Resources & Services Administration. Available online at: https://www.hrsa.gov/opa (Accessed April 4, 2024).

[28] Three Day Payment Window | CMS. Available online at: www.cms.gov https://www.cms.gov/medicare/payment/prospective-payment-systems/acute-inpatient-pps/three-day-payment-window (Accessed April 4, 2024).

